# Richardson on Nitrous Oxyd

**Published:** 1868-09

**Authors:** 


### Editorial.

### RICHARDSON ON NITROUS OXYD.

*Et tu, quoque, Brute!*

THE use of nitrous oxyd having suddenly reappeared as a fierce epidemic, it is not strange that many and curious things are said of it, and that many wild fancies, and absurd theories, in reference to it, are promulgated. But it is strange if Dr. Richardson holds the sentiments with which the *London Lancet* accredits him; and it is equally strange if the *Lancet* seriously misrepresents him.

The following is a quotation from the *Lancet* purporting to give Dr. Richardson's views:—

"It was painful, he remarked, to see the childish excitement with which nitrous oxide and its effects had recently been dwelt on. The gas had been treated as an unknown, wonderful, and perfectly harmless agent; whereas, in simple fact, it was one of the best known, least wonderful, and most dangerous of all the substances that had been applied for the production of general anæsthesia. No substance had been physiologically studied with greater scientific zeal or more rigid accuracy; and no substance had been more deservedly given up as unfit and unsafe for use. It had caused death in the human subject, and on animals it was so fatal that with the utmost delicacy in its use, it was a critical task thoroughly to narcotize an animal with the gas without actually destroying life. In some cases, also, animals died after recovering from the insensibility.

"Respecting the modes of action of the nitrous oxide, Dr. Richardson explained that it was not, in the true sense, the agent that caused the insensibility. It acted indirectly, and the immediate stupefier was really carbonic acid. In fact, nitrous oxide is an asphyxiating agent. There are two explanations of this. It may be that the nitrous oxide quickens the oxidation of blood, and so causes accumulation of carbonic acid in the blood; or it may be—and this is most probable—that it acts by checking the outward diffusion of carbonic acid. The vapor density of nitrous oxide and of carbonic acid is the same—namely, 22, taking hydrogen as unity; and as diffusion of gases into the blood and out of it, is governed by the same laws as in ordinary diffusion, to make an animal breathe nitrous oxide is virtually equivalent to making it breathe carbonic acid itself, the diffusion of carbonic being so determinately impeded. The living phenomena were also in character; the arterial blood was rendered venous by nitrous oxide; the animal temperature fell; the skin became livid. And although these symptoms might be induced many times without actually destroying life, they could not be sustained for any length of time without certain disaster."

As to this being the "best known" of the general anæsthetics, it is enough to say, that neither Doctor R., nor any one else *knows* how to obtain, regularly and uniformly, pure nitrous oxyd. Close approximations are all that has yet been attained. And he tells us, and truly, that he doesn't know how it operates in producing anæsthesia. Like a Yankee, he guesses twice, and then *guesses* that one guess is probably more accurate than the other. And this is the authority which the *Lancet* says, "cannot be questioned."

As to the "rigid accuracy" with which it has been studied, it may be well to remember that all the early experimenters used the gas mixed with other substances. In other words, they administered to the patient *his own breath*, and a mixture of gases, in which nitrous oxyd predominated. Occasionally, they advise us to use large sacks for inhaling—not smaller than an ox's bladder, as Regnault tells us. Just think of a patient breathing out of and into the bladder of an ox!

When first *successfully* used as an anæsthetic, by Dr. Horace Wells, larger bags were used; but still the results were not satisfactory. The want of reliable apparatus, the non-portability of the gas, and the transient anæsthesia produced by it, rendered its supercedure by sulphuric ether quite easy.

As now used, with valved inhalers, and with greater care in the preparation of the gas, the results are radically different; and if properly administered, the symptoms are as unlike those described by Dr. R., as can well be imagined. The arterial blood is not rendered venous. I cannot be mistaken in this. I have repeatedly seen it breathed for twenty minutes, air being all the time excluded, without change of complexion, and without change of color in the arterial blood. The animal temperature *rises* about as often as it falls, perhaps oftener; nor does the skin become livid when pure gas is properly administered. Though constantly using it, I have not seen the skin livid a half dozen times in as many months.

That it has caused death in animals amounts to nothing, as the animals were smothered in their own exhalations. No one claims that carbonic acid is a safe anæsthetic; and it is not improved by its accompaniments, furnished from the perspiration and otherwise. That "it has caused death in the human subject" is doubtful. It is wonderful if it has not; for it is administered by many who know little of its properties and mode of preparation, and still less of the laws of life. And they have used it many times. During the past year, it is probable, the gas has been given 200,000 times in the United States. If any one regards the estimate as too high, he is at liberty to reduce it to his notion. Now, if it were the "most dangerous of all the substances that had been applied for the production of general anæsthesia," it would have killed one of these 200,000; (more or less) but it has not done it. Chloroform has killed several, while, by no means used so frequently as the oxyd.

We do not pretend to notice much that is written on nitrous oxyd. But when Doctor R., speaks he is listened to. It is unfortunate that so many follow authority instead of thinking for themselves. For example, no quantity of nitrous oxyd, tried by Sir H. Davy, as he used it, was found to sustain respiration for a longer period than four minutes. Accordingly authors have told us from that date till nearly the present, that four minutes is the limit.

Those who breathe the gas properly do not find it an asphyxiating agent. I once breathed it an hour, (taking less than a dozen inspirations of atmospheric air during the time) and the pulse did not vary four beats from seventy at any time, it being carefully examined every five minutes. The complexion was natural at the close of the experiment, the temperature was comfortable, and that evening, and the next day, I attended to business as if nothing had happened.

It is, indeed, very strange how little the oxyd affects the action of the heart. I have, in hundreds of cases, seen the respirations reduced to eight, six or even to three to the minute, while the pulse remained perfectly natural.

It was not the intention to be surprised into writing on nitrous oxyd until ready to do so at length ; but respect for Dr. R., and regret that he is so far astray on the subject, has induced a change of programme. With the hope and trust that there is an unintentional misrepresentation of Dr. R's views in the editorial referred to, the subject is dropped for the present. W.

